# The Adverse Effects of Air Pollution on the Nervous System

**DOI:** 10.1155/2012/782462

**Published:** 2012-02-19

**Authors:** Sermin Genc, Zeynep Zadeoglulari, Stefan H. Fuss, Kursad Genc

**Affiliations:** ^1^Department of Neuroscience, Health Science Institute, Dokuz Eylul University, Inciralti, 35340 Izmir, Turkey; ^2^Department of Molecular Biology and Genetics, Bogazici University, 34342 Istanbul, Turkey

## Abstract

Exposure to ambient air pollution is a serious and common public health concern associated with growing morbidity and mortality worldwide. In the last decades, the adverse effects of air pollution on the pulmonary and cardiovascular systems have been well established in a series of major epidemiological and observational studies. In the recent past, air pollution has also been associated with diseases of the central nervous system (CNS), including stroke, Alzheimer's disease, Parkinson's disease, and neurodevelopmental disorders. It has been demonstrated that various components of air pollution, such as nanosized particles, can easily translocate to the CNS where they can activate innate immune responses. Furthermore, systemic inflammation arising from the pulmonary or cardiovascular system can affect CNS health. Despite intense studies on the health effects of ambient air pollution, the underlying molecular mechanisms of susceptibility and disease remain largely elusive. However, emerging evidence suggests that air pollution-induced neuroinflammation, oxidative stress, microglial activation, cerebrovascular dysfunction, and alterations in the blood-brain barrier contribute to CNS pathology. A better understanding of the mediators and mechanisms will enable the development of new strategies to protect individuals at risk and to reduce detrimental effects of air pollution on the nervous system and mental health.

## 1. Introduction

Air pollution collectively describes the presence of a diverse and complex mixture of chemicals, particulate matter (PM), or of biological material in the ambient air which can cause harm or discomfort to humans or other living organisms. The sources of air pollution can either be natural (e.g., volcanic eruptions) or manmade (e.g., industrial activities), and air pollution emerges as a serious health problem especially in rapidly growing countries. Millions of people worldwide are chronically exposed to airborne pollutants in concentrations that are well above legal safety standards [1]. Therefore, morbidity and mortality attributable to air pollution continue to be a growing public health concern worldwide. Air pollution ranks eighth among the leading risk factors for mortality and accounts for 2.5% of all deaths in developed countries [2]. The World Health Organization (WHO) estimates that air pollution is responsible for over 3 million premature deaths each year [3]. Epidemiological and observational studies identified a strong link between the exposure to contaminants in the ambient air and adverse health outcomes, such as hospitalization and mortality [4]. Exposure to air pollutants has been associated with marked increases in cardiovascular disease morbidity and deaths resulting from myocardial ischemia, arrhythmia, heart failure, and respiratory diseases such as lung cancer and asthma [3, 4].

About a decade ago, the central nervous system (CNS) has also been proposed to be a target organ for the detrimental effects of airborne pollutants [5]. Indeed, emerging evidence from recent epidemiological, observational, clinical, and experimental studies suggest that certain neurological diseases, such as Alzheimer's disease (AD), Parkinson's disease (PD), and stroke, may be strongly associated with ambient air pollution.

Mechanistically, air pollution may affect the nervous system through a variety of cellular, molecular, and inflammatory pathways that either directly damage brain structures or lead to a predisposition to neurological diseases. Although ischemic stroke (chronic exposure to ambient air pollution), multiple sclerosis (MS, exposure to second-hand smoking), and PD (manganese content in the ambient air) are currently the only neurological disorders for which a strong link to ambient air pollution has been established, it is not unlikely that other CNS disorders are also attributable to air pollution [6–8].

It has been suggested from epidemiological and observational studies that exposure to airborne pollutants can contribute to neurodegenerative disease processes already from early childhood on, especially if the individuals are chronically exposed to the contaminants [1, 9–11]. Air pollutants affect the CNS either directly by transport of nanosized particles into the CNS or secondarily through systemic inflammations. Either of the effects can be caused by the physical characteristics of the particle itself or by toxic compounds that adsorb on the particles [12, 13]. Although the exact mechanisms underlying brain pathology induced by air pollution are not fully understood, several lines of current evidence point out that neuroinflammation, oxidative stress, glial activation, and cerebrovascular damage might be the primary pathways [1, 14].

In this paper, we provide an overview of the different classes of air pollutants and their potential ways to entry by which they could get into contact with the CNS. We summarize findings of epidemiological, observational, clinical, and experimental studies which describe a link between air pollution and neurological diseases or neurodevelopmental disturbances. Finally, we summarize the current understanding of the adverse effects of air pollutants on the nervous system and mental health on a cellular and molecular level.

## 2. Components of Air Pollution

Air pollution represents a diverse mixture of substances including PM, gases (e.g., ground-level ozone, carbon monoxide, sulfur oxides, and nitrogen oxides), organic compounds (e.g., polycyclic aromatic hydrocarbons and bacterial endotoxins), and toxic metals (e.g., vanadium, lead, nickel, copper, and manganese) that can be found in outdoor and indoor air [1, 15]. Among these, PM and ground-level ozone, which are formed primarily from nitrogen oxides and volatile organic compounds, appear to be the most widespread and harmful components. Of those, PM is especially relevant for nervous system damage and can be found as a mixture of solid particles and liquid droplets, that are suspended in the air [1]. Most individual components of atmospheric PM are not especially dangerous and some major constituents, such as sodium chloride, are harmless [16].

PM is characterized by its size and aerodynamic property which is directly related to its biological effects. For instance, only particles less than 10 *μ*m in diameter can be inhaled deep into the lungs, whereas larger particles usually get trapped in the upper airways. Generally, coarse particles with an aerodynamic diameter of 2.5 to 10 *μ*m (PM_10_), fine particles of less than 2.5 *μ*m (PM_2.5_), and ultrafine (UFPs), or nano-sized (NP) particles of less than 0.1 *μ*m can be classified [15, 17].

Road and agricultural dust, tire wear emissions, products of wood combustion, construction and demolition works, and mining operations are the primary sources of PM_10_. PM_2.5_ particles commonly originate from oil refineries, metal processing facilities, tailpipe and brake emissions, residential fuel combustion, power plants, and wild fires [15]. They are formed from gas and condensation of high-temperature vapors that are formed during combustion and industrial activities. PM_2.5_ can be composed of both organic and inorganic compounds, including sulfates, nitrates, carbon, ammonium, hydrogen ions, lipopolysaccharide (LPS), metals, and water [1]. Diesel exhaust particles (DEPs), however, are the major components found among ambient fine particles.

UFPs are mostly combustion-derived NPs, which can be produced by internal combustion engines, power plants, incinerators, and other sources of thermodegradation. They can carry soluble organic compounds, polycyclic aromatic hydrocarbons, and oxidized transition metals on their surface [18]. UFPs have distinct features that render them more dangerous than other PMs. For instance, they have been shown to inhibit phagocytosis and to stimulate inflammatory responses [16]. Although the effects of UFPs have been studied less extensively than those of PM_2.5_ and PM_10_, there is evidence that the size of the particles is negatively correlated with their adverse health effects [19].

Indeed, ambient UFP concentrations are found to be directly correlated with mortality [20]. Current national air quality standards are based on the mass concentration of PM. However, when compared to fine particles at similar mass concentrations in the air, UFPs are much more numerous and have a larger combined surface area, enhanced oxidant capacity, greater inflammatory potential, and higher pulmonary deposition efficiency [16, 17, 21, 22]. A major risk of UFPs arises from the fact that they are not filtered out during their passage through the nose and bronchioles but are able to penetrate deep into the lung where they eventually enter the blood circulation and can get distributed throughout the body.

## 3. Entry of Air Pollutants into the Central Nervous System

**Figure 1:**
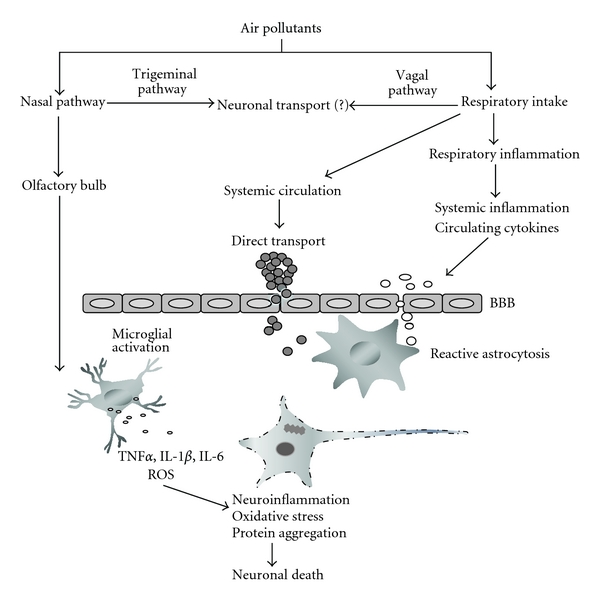
The impact of air pollution on the brain through multiple pathways.

Sustained exposure to significant levels of airborne UFPs, PM, and LPS may result in the direct translocation of these pollutants to the CNS where they can result in neuropathology through a variety of pathways and mechanisms (Figure 1). Alternatively, air pollutants might not enter the CNS directly, but could exert adverse effect on the CNS by triggering the release of soluble inflammatory mediators from primary entry organs or secondary deposition sites. The release of inflammatory agents could then lead to or alter the susceptibility for neuroinflammation and neurodegeneration in the CNS.

Once taken up by the body, fine PM or NPs could rapidly enter the circulatory system with the potential to directly affect the vascular system. For instance, NPs could be inhaled and cross the alveolar-capillary barrier in the lungs. The ability of NPs to cross this barrier is influenced by a number of factors that include the size of the particles, their charge, their chemical composition as well as their propensity to form aggregates. Even though the translocation of inhaled or instilled NPs across the alveolar-capillary barrier has been clearly demonstrated in animal studies for a range of NPs [23, 24], it has been more difficult to directly demonstrate this mechanism in humans [3].

Regardless of the route of entry, NPs that reach the circulation could directly affect vascular endothelium cells by creating local oxidative stress or by causing proinflammatory effects similar to those seen in lung tissue. Inflammatory mediators that are produced in the respiratory tract as a consequence of chronic pollutant-induced epithelial and endothelial injury can lead to systemic inflammation [25]. The systemic inflammation is accompanied by the production of proinflammatory cytokines such as tumor necrosis factor alpha (TNF*α*), interleukin-6 (IL-6), and interleukin-1beta (IL-1*β*), for which blood vessels in the brain exhibit constitutive and induced expression of receptors [1, 26]. The cytokines could thus activate cerebral endothelial cells, disrupt the blood-brain barrier (BBB) integrity, or trigger signaling cascades that lead to the activation of mitogen-activated protein (MAP) kinase, and nuclear factor kappa B (NF*κ*B) transcription factor-mediated pathways. Disruption of the BBB could then be followed by trafficking of mast cells and inflammatory cells expressing CD163, CD68, and HLA-DR to the damaged site [10]. In addition, circulating cytokines that are released by inflamed peripheral organs or endothelial cells could stimulate peripheral innate immune cells, activate peripheral neuronal afferents, or enter the brain by diffusion and active transport thereby worsening the condition synergistically [27, 28]. Accordingly, brain tissue samples from individuals residing in highly polluted areas show an increase in the number of infiltrating monocytes or activated microglia, increased expression of IL-1*β*, BBB damage, endothelial cell activation, and brain lesions in the prefrontal lobe [10, 11].

Airborne LPSs may induce neuroinflammatory responses directly by activating the brain's innate immune system. The effect of LPS on neuroinflammation is well studied in a bacterial endotoxin/LPS-based experimental model of PD that constitutes an important tool to delineate the mechanisms of neuroinflammation-mediated loss of dopaminergic neurons [29]. This system could also be exploited in combination with exposure to other environmental toxins and air pollutants. Brain uptake of circulating LPSs is usually low, and most effects of peripherally administered LPS are likely to be mediated through LPS receptors located outside the BBB [30]. Thus, LPSs might stimulate afferent nerves, act at circumventricular organs, or alter the permeability of the BBB. Circumventricular organs are specialized brain structures located around the third and fourth ventricle. They are highly vascularised and lack a BBB; therefore, they allow for a direct uptake of chemicals circulating in the blood stream by neuronal cells [31].

The very small UFPs on the other hand easily penetrate cell membranes because of their large surface-to-volume ratio, which also enables them to traverse the classical barriers in the lung and the brain. Their ability to cross cell membranes easily explains why PM can be found inside neurons or erythrocytes [1, 32]. It has also been proposed that the close contact between endothelial cells and erythrocytes could represent a route for the exchange of PM between activated endothelial cells and UFP-loaded erythrocytes [1, 33, 34].

Another important and more direct route for UFPs to enter the nervous system is through the olfactory mucosa, which is a neuronal epithelium that is in direct contact with the environmental air [35–37]. Thus, fine and UFPs may reach the brain through olfactory receptor neurons or the trigeminal nerve. Olfactory receptor neurons are bipolar sensory neurons that mediate the sense of smell by conveying sensory information from the nose to the CNS. The olfactory epithelium is covered by a layer of sustentacular cells, but olfactory sensory neurons extend their dendrites into the mucous layer covering the olfactory epithelium where they directly interact with odorants inhaled with the air. Nasally inhaled pollutants that reach the olfactory mucosa could enter the cilia of olfactory receptor neurons by pinocytosis, simple diffusion, or receptor-mediated endocytosis. Once incorporated into sensory neurons, they could be transported by slow axonal transport along the axons to the olfactory bulb [38]. From there, pollutants could be transported further into the CNS along mitral cell axons that project from the olfactory bulb to multiple brain regions, including the olfactory cortex, the anterior olfactory nucleus, the piriform cortex, the amygdale, and the hypothalamus.

Accordingly, UFPs have been observed in human olfactory bulb periglomerular neurons and trigeminal ganglia capillaries [10]. Similarly, a decreasing gradient of metal (vanadium and nickel) deposition and accompanying tissue damage from the nose to the brain has been reported in the canine nervous system, confirming the importance of the nasal route for the entry of air pollutants into the CNS [39]. Controlled exposures of rats to UFPs and metals also demonstrated their accumulation in the olfactory bulb [40–42]. Taken together, these findings suggest that NPs can be taken up directly by the olfactory mucosa and enter the CNS or the cerebrospinal fluid by bypassing the circulatory system [12]. Uptake through the nose might even be enhanced by additional pollutant-induced systemic inflammation by deteriorating the olfactory mucosal barrier, which would result in increased neuropathology.

Additional direct neuronal entry routes for NPs have been described that involve the retrograde and anterograde transport in axons and dendrites such as the transport of inhaled NPs to the CNS via sensory nerve fibers that innervate the airway epithelia [12]. Ground-level ozone exposure activates the CNS through the vagal nerves without the involvement of the thoracic spinal nerves [43]. PM-related LPS is likely to play an important role in these pathways, as shown by vagal upregulation of CD14 [44].

Even though the translocation rate of NPs from their site of entry to secondary organs might be rather low, continuous or chronic exposure to NPs may result in their accumulations in the brain as a secondary target organ in significant amounts [12]. Thus, it is also important to obtain data on the retention characteristics of NPs in both primary and secondary target organs, including associated elimination and clearance pathways [12]. With regard to the CNS, no data on NP elimination are available yet. It is conceivable, however, that CSF circulation provides an excretory pathway for NPs that enter via neuronal uptake. Usually, the CSF serves as a fluid cushion for the brain, but also distributes substances to all brain regions and acts as an elimination route for metabolic waste products [45]. NPs could be eliminated from the CSF through the same mechanisms: uptake of CSF by the blood circulatory system through arachnoid vili or via the nasal lymphatic system. The exact details of NP clearance from the brain, however, await future investigation [12].

## 4. Air Pollution and Neurological Disease

Results about the direct effects of air pollutants and airborne particles on neuronal cells have been reported from experimental studies *in vitro*, using cell culture systems and *in vivo*, using inhalation and instillation paradigms in rodents as well as from epidemiological and controlled clinical studies in humans.

### 4.1. Experimental Studies

#### 4.1.1. *In Vitro* Studies

A variety of *in vitro* studies assessed the potential toxic effects of air pollutants (Table 1), by measuring changes in cell viability, alterations of apoptosis, the dysfunction of mitochondria, the production of reactive oxygen species (ROS), or the production of pro-inflammatory cytokines as sensitive identifiers [1]. Varying degrees of proinflammatory- and oxidative stress-related cellular responses and decreased cell viability were reported upon stimulation with laboratory-generated or filter-collected ambient air particles in different cell culture systems [42]. Of particular interest are studies utilizing neuronal and microglial cell lines or primary cultures of those cells that were exposed to concentrated ambient air particles (CAPs), diesel exhaust particles (DEPs), toxic gases, such as ozone, bacterial endotoxins, such as LPS, or toxic elements, such as manganese. All investigated neuronal, glial or cerebral endothelial cell types were shown to be targets of the toxic effects of air pollutants [46–48]. However, the underlying mechanisms could be rather complex, and some insight into the interaction of different cell types was derived from coculture systems. For instance, it was shown that the neurotoxic effects of DEPs on dopaminergic neurons could be either direct or indirect via the release of inflammatory mediators and ROS from activated microglial cells [46, 49]. Interestingly, pretreatment of neuron-glia cocultures with LPS increased the vulnerability of the cells to the toxic effects of DEP, while DEPs alone were not harmful [49].

An important aspect of *in vitro* toxicity studies is the establishment of dose-response relationships. For instance, low concentrations (20–40 *μ*g/mL of gas per mL of complete medium) of oxygen-ozone were not toxic to astroglial cells, while higher concentrations (60 *μ*g/mL) severely decreases cell viability [48]. Transcriptomic and proteomic profiling of cultured cells upon exposure to CAPs may provide insights into alterations of gene and protein expression. One such study demonstrated the upregulation of inflammatory and innate immunity pathway components in mouse immortalized BV2 cells when exposed to CAPs [50]. Likewise, the expression profiles of microRNAs, which emerged as crucial mediators of posttranscriptional gene regulation, might change during exposure to air pollutants [51]. Indeed, hexahydro-1,3,5-trinitro-1,3,5-triazine (RDX), a common environmental contaminant and explosive nitroamine that is widely used in military ammunition, has been shown to change brain microRNA expression in exposed mice [52].

The rapidly growing number of engineered nanoparticles (ENPs) and nanomaterials (NMs) might also contribute to air pollution as new nanotechnologies are constantly developed, and NMs are used increasingly in daily life through the advent of new products. In addition, ENPs are extensively tested for their usefulness in medical diagnostic and therapeutic applications. Although no human ailments have been directly attributed to NMs so far, preliminary experimental studies indicate that NMs could initiate adverse biological responses and that NPs could have toxicological properties [53]. Thus, ENPs constitute a novel neurotoxic risk and several *in vitro* studies could demonstrate adverse effects of ENPs on CNS cells (not included in Table 1). For instance, titanium dioxide, aluminum oxide, and nanosized silica particles were shown to decrease cell viability and to increase apoptosis in neuronal and endothelial cell cultures [54–58]. These substances also increased the amount of ROS, which resulted in concomitant activation of microglia [54–59]. An important point in *in vitro *nanoneurotoxicity studies is therefore the necessity to accurately characterize particle size, as particles of different size might exert different effects or similar effects to different degrees. In addition, a controlled investigation of the physicochemical properties of the NPs over time and their interactions with culture media should also be considered [60, 61]. Although NPs in environmental air samples might be much more heterogeneous, epidemiological and toxicological studies with airborne ultrafine particles can be viewed as the basis for the expanding field of nanotoxicology [42].

*In vitro* studies bear several distinct advantages for studying neurotoxic effects of air pollutants because the technology is cheap, the cultured cells grow rapidly, and the assays provide reproducible results. However, many times immortalized cell lines are used, which might not correctly reflect the more complex responses of native CNS cells or of neurons in their natural complex environment. Unfortunately, long-term and large-scale cultures of primary CNS cells are still challenging and thus might not be useful for high-throughput screening of toxicological effects. The emerging field of induced pluripotent stem cells, which can be easily derived from somatic cells such as dermal fibroblasts and keratinocytes, may provide a solution to this problem and induced pluripotent stem cells could soon emerge as a novel experimental paradigm for human neurotoxicity studies [62, 63].

Despite their advantages, *in vitro* studies have also important limitations, some of which are methodological. The interpretation and cross-comparison of results from different research groups might be hampered because of the use of particles with different chemical compositions or different culture cells. The duration of exposure and concentrations might differ across laboratories. More importantly, however, responses of cultured cells might not faithfully reflect the responses of the entire body system or target organ. In general, ultraphysiological doses of air pollutants are used in cell cultures studies and the long-term study of the effect of chronic exposure to low doses of potentially toxic material is not feasible. Organotypic cell cultures and tissue explant cultures might be more useful in this regard since the integrity of tissue of interest is fully or partially preserved. Because systemic effects and biodistribution of air pollutants cannot be investigated in *in vitro* assays, *in vivo *studies provide additional and important information on the adverse effects of air pollutants.

#### 4.1.2. *In Vivo* Studies

The confirmation of *in vitro* results through realistic *in vivo* studies is mandatory to validate hypotheses generated from *in vitro* studies [12]. *In vivo* studies are invaluable tools for the examination of bio-distribution, the biokinetic properties, and the pathophysiological effects of air pollution on the whole body system. They also provide an opportunity to study neurobehavioral effects of air pollution in intact living animals. Novel noninvasive imaging techniques can be used to visualize neuroinflammation, microglia activation, brain redox-status, and BBB integrity in live animals [64, 65]. Importantly, *in vivo* studies allow the use of experimental conditions, routes of administration, and exposure regimes that are not available in cell culture systems. For instance, they enable a comparison of the effects of acute, subchronic, and chronic exposure of the whole animal. Likewise, pollutants can be administered through different natural and artificial routes such as inhalation, nasal and intratracheal instillation, or intraperitoneal injection (Table 2). Like cell culture studies, whole animal studies are amenable to investigate alterations in gene and protein expression, and activation of signaling pathways upon exposure to air pollutants. Finally, prevention strategies and therapeutic approaches can be tested in a preclinical setting.

To investigate the effect of certain gene products on the susceptibility to damage by air pollutants, genetically modified animals can be used. For instance, one study used Apolipoprotein E (ApoE) knockout (ApoE^−/−^) mice and could show that ApoE deficiency enhances air pollutant-induced neurotoxicity [66]. Exposure to UFPs activates NF*κ*B and AP-1 transcription factors via JNK-activation in ApoE^−/−^ mice in a dose- and duration-dependent manner [67]. In a more recent study, these findings were confirmed, providing evidence that air pollution can produce neuropathological damage in individuals that are susceptible to oxidative stress [68].

Tin-Tin-Win-Shwe et al. used wild-type male BALB/c mice and instilled carbon black (CB) intranasally [74]. Six hours after instillation, the mice were intraperitoneally injected with the bacteria cell wall component lipoteichoic acid (LTA) and the authors could show that LTAtreatment potentiates CB-induced neurological effects. CB modulates the levels of extracellular amino acid neurotransmitter and proinflammatory cytokine IL-1*β* mRNA expression synergistically with LTA in the mouse olfactory bulb. In a recent study by Zanchi et al., rats were exposed to residual oil fly ash (ROFA), one of the residues generated by combustion, by intranasal instillation and were treated with the antioxidant N-acetylcysteine (NAC) intraperitoneally for 30 days [73]. ROFA instillation alone induced an increase in lipid peroxidation levels in the striatum and the cerebellum, whereas NAC treatment had preventive effects.

Ozone is by far the most important air pollutant in terms of its concentration, its persistence, and its ubiquitous occurrence. A list of preclinical studies that investigated the neurotoxic effects of ozone inhalation using different experimental paradigms is given in Table 2. For instance, Pereyra-Muñoz et al. showed that chronic (4 h daily for 15 or 30 days) and low-dose (0.25 ppm) exposure induces oxidative damage to neurons in the striatum and substantia nigra [75]. Angoa-Pérez et al. exposed ovariectomized rats to air loaded with ozone for 7, 15, 30, or 60 days (0.25 ppm, 4 h per day) [76]. A second experimental group of ovariectomized rats were treated with 17[beta]-estradiol 2 h after ozone exposure in an otherwise identical exposure regime. The data suggest that chronic ozone inhalation produces oxidative stress and loss of dopaminergic neurons in the substantia nigra and that the effects can be reduced by treatment with 17[beta]-estradiol [71, 76]. Neural mechanisms underlying adaptive responses to acute ozone exposure were also studied in adult rats that were subjected to 0.5 ppm ozone exposure for 3 h and were then allowed to recover for 3 h before examination. In this paradigm, acute ozone exposure had an effect primarily on glial cells in the CNS [79]. The protein expression levels of vascular endothelial growth factor (VEGF) were upregulated in central respiratory areas, such as the nucleus tractus solitarius (NTS) and the ventrolateral medulla (VLM). Persistent VEGF upregulation following ozone exposure may contribute to brain repair and consecutive functional adaptations. Rats that inhaled 0.5 or 2 ppm ± 10% of ozone for 1.5–120 h suffered from lung inflammation that induced the activation of NTS neurons through the vagus nerve. It also promoted neuronal activation in other, stress-responsive regions of the CNS as could be demonstrated by up-regulated levels of the immediate early-gene product c-Fos [43].

As exemplified above, *in vivo* studies offer a unique possibility to test the potential of neuroprotective agents such as hormones and antioxidants against air pollutants [71, 73, 76, 78]. Selective inhibitors of the cyclooxygenase-2 (COX-2) enzyme have been tested in young healthy dogs which were residents of highly air polluted urban regions. Inhibition of COX-2 showed beneficial effects probably by reducing frontal lobe IL-1*β* expression [80]. Interestingly, treatment with dark chocolate has also been found to be neuroprotective against long-term air pollution in mice [44].

Despite the clear advantages of *in vivo* studies that were summarized here (studying pathophysiological mechanisms or neurobehavioral responses and testing preclinical preventive and treatment strategies), a long list of confounding parameters experimentally may obscure the results. Methodological details such as sex, age, strain, dose, and the particular assay that was used to measure the outcome should be considered carefully when comparing results across different studies. In particular body size, age, gender, species, and strain are known to have dosimetric effects in air pollution research [81]. Although there is growing epidemiologic evidence that associations between air pollution and respiratory health differ between females and males, comparative studies or studies on female rodents in general are limited [72, 82].

Likewise, only a single study evaluated the influence of age on air pollution-induced CNS injury [78]. In this study, ozone inhalation resulted in high-lipid peroxidation in the frontal cortex of old rats, which is in contrast to findings in young rats, where oxidative stress injury occurred in the hippocampus. Region specific inflammation and alterations in gene expression were also seen after DEPs exposure, suggesting a selective vulnerability of specific neuronal subpopulations similarly to the selective loss of specific neurons that is typical for certain neurodegenerative diseases [69, 83]. Although strain difference is an important variable in a variety of lung injury studies, it is a widely neglected parameter in air pollution-induced CNS injury research [84, 85].

Variations in the geographic location of sample collection, and seasonal climate variations during the collection of ambient air samples are neglected oftenly as well. However, these parameters have a crucial impact on the results and should be clearly described in all studies. Use of filtered ambient air samples may, on one hand, simulate real-world exposure conditions, on the other hand, the samples also contain unidentified or unmeasurable components. Thus, the inherent heterogeneities of *in vivo *experimental paradigms show a need for standardization of test parameters that enables a more reliable comparison between studies from different laboratories. The lack of such a standardized system also hampers the translation of data from preclinical studies to humans. In particular, the anatomy of the respiratory tract and the nasal cavity, the breathing pattern (nasal breathing is obligatory for rodents), and brain anatomy differ greatly across species and impede generalization of the results. For instance, while the olfactory mucosa lines more than 50% of the surface of the nasal cavities in rodents, the human olfactory tissue is restricted to a mere 3–5%. The use of nonhuman primates would provide results more relevant to humans, but poses great ethical concerns.

### 4.2. Epidemiological, Postmortem, and Clinical Studies

#### 4.2.1. Stroke

While cardiorespiratory effects of air pollution have been extensively investigated [3], only preliminary findings are available on the effects of airborne pollutants on the CNS. Stroke is one of the most prevalent CNS disorders which can be caused by air pollution. A relationship between air pollution and stroke was first reported after the Great London fog [8], but similar results were obtained from different geographic regions that include Canada, Japan, Italy, Sweden, USA, UK, France, Taiwan, and Korea [86–95]. However, a one-to-one comparison of these studies is difficult because each study measured different pollutants, investigated populations with different genetic background, or people exposed to different environmental conditions, in addition to evaluating different stroke-related parameters. Despite the experimental differences, a large number of studies demonstrated a positive correlation between stroke mortality rates, hospital admission, and outdoor pollution [87–90, 92–95], although contradictory results were reported as well [91, 96]. Interestingly, a Canadian study showed that only a specific subgroup of patients, those suffering from diabetes mellitus, was at high risk for ischemic stroke [91]. Age and gender may also differentially affect the risk of air pollution-related ischemic stroke. Elderly people and women appear to be more sensitive to the effect of air pollutants [87]. It also appears that the air pollution-related ischemic stroke risk is higher than the risk for hemorrhagic stroke [8, 86]. Hemorrhagic and ischemic strokes have distinct pathogenesis and also differ in terms of other risk factors.

Mechanistically, the correlation between air pollution and stroke might be due to the observation that fine PM and UFPs exert procoagulant effects *in vivo *[97, 98]. Yet, the stroke risk increases with both, short-term and long-term exposure to outdoor air pollution [90, 99], although the effects of long-term exposure on stroke risk are less prominent [99]. In addition to these epidemiological findings, a limited number of *in vivo* studies also support a close correlation between air pollution and stroke. For instance, SO_2_ inhalation caused cerebral changes similar to the alterations resulting from middle cerebral artery occlusion (MCAO) and aggravated histological changes in ischemic brain regions [100].

Air pollution will continue to become a major health problem, especially in developing countries and rapidly growing economies. Unfortunately, booming economic development increases air pollution and related disease including stroke. Thus, there is a great demand to organize population-based and prospective studies to evaluate and to develop preventive measures against the unfavorable effect of air pollution on severe cerebrovascular diseases, such as ischemic stroke.

#### 4.2.2. Neurodegenerative Diseases

Concomitant with a general increase in life expectancies worldwide, the incidence and prevalence of common neurodegenerative diseases grow as well, thereby increasing the financial and social burden on individuals and society. Alzheimer's disease (AD), the most prevalent neurodegenerative disease, is characterized by extracellular deposition of amyloid-beta (A*β*) peptide fibrils known as amyloid plaques and intracellular protein aggregates called neurofibrillary tangles (NFTs) [101]. AD is the most common cause of dementia in aged people, affecting 27 million people globally. Parkinson's disease (PD), the second common neurodegenerative disorder, is caused by the degeneration of dopaminergic neurons in the substantia nigra and a progressive loss of dopaminergic neurotransmission in the caudate and putamen of the neostriatum [102]. This severe movement disorder affects 1-2% of the population above the age of 50. Most AD and PD cases are sporadic, and age is the leading risk factor. The etiologies of the diseases, however, are multifactorial, and the risk factors include environmental factors and genetic predisposition. Environmental exposures to metals, air pollution, and pesticides as well as nutritional factors are common risk factors for neurodegenerative diseases [103]. Although different neurodegenerative diseases have distinct pathologies and clinical presentations, they often share common mechanisms such as protein aggregation, oxidative stress injury, neuroinflammation, microglial activation, apoptosis, and mitochondrial dysfunction, which ultimately result in the loss of specific neurons [101, 102]. Accumulating evidence suggest that exposure to air pollution can trigger these common denominators of neurodegenerative diseases and lead to neuropathology.

The first histopathological evidence for a link between air pollution and neuropathology came from studies that were carried out on animal populations that are naturally exposed to polluted urban environments in Mexico City [1]. Using light and electron microscopy, Calderón-Garcidueñas et al. reported significant inflammatory and neurodegenerative changes in the olfactory mucosa, the olfactory bulb as well as in subcortical and cortical structures in otherwise healthy mongrel canines, whereas similar changes were not evident in control groups inhabiting less-polluted rural areas [104]. Breakdown of nasal and olfactory barriers, alterations in the BBB, and degeneration of cortical neurons were observed even in animals that were younger than 1 year of age. With growing age, and therefore extended exposure, the dogs exhibited reactive astrogliosis, white matter glial cell apoptosis, ApoE immunoreactivity in vascular cells, and nonneuritic plaques and NFTs. These findings suggest an accelerated AD-like neuropathology in chronically exposed animals. Feral dogs naturally exposed to urban air pollution also showed DNA damage in olfactory and hippocampal genomic DNA [39]. Cerebral inflammatory responses were associated with the neurohistological findings as demonstrated by nuclear translocation of the neuronal NF*κ*B p65 subunit, increased inducible nitric oxide synthase (iNOS) immunoreactivity in endothelial, glial and neuronal cells, and increased endothelial and glial COX-2 immunoreactivity [39, 104]. Animals from polluted areas exhibited deposits of diffuse amyloid plaques a decade earlier than control animals from less-polluted regions [39, 104]. Although most animals do not develop the full human pathology of AD, aged dogs are known to suffer from cognitive dysfunctions that resemble key aspects of AD [105]. The decline in executive functions and the impairment of learning and memory represent a spectrum that comprises normal aging, mild cognitive impairment, and early/mild AD in humans [106]. However, dense core neuritic plaques and NFTs could not be observed consistently in the dogs. Because of the numerous atmospheric contaminants found in the highly polluted air of Mexico City, postmortem studies on resident feral dogs could only link the neuropathology to the complex mixture of ozone, PM, LPS, and unmeasurable air pollutants [14]. Thus, whether airborne UFPs are causatively involved in the observed CNS change remain to be determined [5, 16]. However, the oil-combustion PM-associated metals nickel and vanadium, as well as UFPs were detected in the dogs brains, indicating that brain uptake of metals and UFPs may occur in natural exposure settings [11, 39].

Similar findings were recently observed in postmortem examinations of human samples and in laboratory animals [1, 14]. Adult human residents of highly polluted urban areas of Mexico City exhibit significantly higher COX-2 expression in the olfactory bulb, the hippocampus, and the frontal cortex, and greater neuronal astrocytic accumulation of A*β*_42_ when compared to age-, gender-, and education-matched subjects from cities with low pollution levels [9, 13]. Based on evaluation of the clinical medical records and information from relatives and coworkers by 2 physicians, each subject was considered cognitively and neurologically fit when alive [9]. The neuropathology, however, could be observed in subjects as early as in the second decade, suggesting that neuropathologies induced by chronic exposure to high levels of air pollution share similarities with the pathology of AD [107]. Although NFTs or A*β* neuritic plaques could not be observed because of the relative young age of the subjects, neuroinflamation and intraneuronal A*β*_42_ accumulation in target brain areas may be compatible with a premature accelerated process preceding AD neurodegeneration. Most interestingly, a recent postmortem study on children and young adults who died suddenly has shown that lifelong exposure to air pollution is associated with neuroinflammation, altered innate immune responses, disruption of the BBB, endothelial activation, and accumulation of disease proteins (A*β*_42_ and *α*-synuclein) in the CNS [10]. Moreover, A*β*_42_-immunoreactivity was higher in brain tissue derived from carriers of the ApoE  *ε*4 allele than those of ApoE *ε*3 carriers suggesting that a specific genotype constitutes a higher risk for developing AD in a polluted environment. The ApoE *ε*4 allele is known to contribute to a genetic predisposition for late-onset AD, although the mechanisms by which ApoE *ε*4 influences onset and progression of the disease are not well understood [101, 108].

The accumulation of *α*-synuclein in the brain of young people that were exposed to air pollution lifelong is noteworthy [10]. *α*-synuclein is a major component of Lewy bodies, a pathological hallmark of PD [102]. Dopaminergic neurons were found to be selectively vulnerable to DEPs both *in vitro* and *in vivo *[46, 49]. However, a recent epidemiological study from Canada did not support a direct link between the markers of traffic-generated air pollution and PD, although an association between ambient manganese pollution and the risk of physician-diagnosed PD was reported [109].

A further interesting similarity between air pollution-induced neuropathologies and neurodegenerative disorders is the early involvement of the olfactory bulb [110]. Olfactory dysfunction, especially in ApoE *ε*4 carriers, can be seen from childhood in individuals that grew up in highly polluted environments. Yet, olfactory dysfunction is also among the first clinical signs of AD and PD [111]. In sporadic PD, olfactory impairment precedes motor symptoms by years and is independent of the loss of dopaminergic neurons. In AD, however, olfactory dysfunction and disease progression correlate [112].

Recent epidemiological studies combined with psychological tests support an association between chronic exposure to traffic-related air pollution and decreased cognitive function in both genders [113, 114]. Altogether, these findings warrant further and more extensive epidemiological, forensic, and toxicological studies that aim to understand the association between chronic exposure and the risk of neurodegenerative diseases development. Such efforts may lead to the development of preventative strategies for these devastating diseases in certain risk groups.

### 4.3. Implications for Neurodevelopment and Mental Health

Normal brain development is a complicated process that involves controlled cell proliferation, neuronal migration from their place of birth to their final location, and the establishment of specific connections between neurons and target tissues [115]. All of these processes are tightly controlled, but are also influenced by environmental conditions. Air pollutants can affect the brain at any age, but the developing brain is particularly vulnerable because of its high neuronal proliferation and differentiation rates and its immature metabolism and imperfect BBB [116]. Disturbances of developmental processes in the brain can lead to permanent abnormalities that translate into later life.

In developing embryos, the placenta serves as a barrier against many environmentally hazardous substances, but it might not be protective against all components of air pollution. Among documented hazards that affect neurodevelopment are certain industrial chemicals, maternal smoking, alcohol, certain drugs, noise, diet as well as maternal stress [117]. This section, however, only focuses on the effects of air pollutants on neurodevelopment.

Ozone is one of the best studied substances in preclinical examinations that assess the effects of exposure to an air pollutant during the prenatal period. Prenatal ozone exposure leads to permanent damage of the cerebellum [118] and disruption of the cerebellar monoaminergic system [119]. In addition, prolonged prenatal ozone exposure altered the levels neurotrophic factor in the brain. CD-1 rats showed reduced nerve growth factor levels in the hippocampus and increased brain-derived neurotrophic factor levels in the striatum when exposed to ozone [120]. Changes in neuronal responses and neuronal injury were also evaluated by immunohistochemistry in rats using c-Fos immunolabeling as a marker for neuronal activity and tyrosine hydroxylase labeling to highlight catecholaminergic neuron injury. Ozone exposure during the prenatal period induced long-lasting changes in the nucleus tractus solitarius (NTS), important respiratory control center [121].

*In vivo* studies have shown that prenatal exposure to DEPs can also affect brain development [122, 123]. *In utero *administration of a low dose of DEPs (1.0 mg DEP/m^3^) reduced locomotor activity and dopamine turnover in the striatum [122] and affected monoamine metabolisms in a variety of brain regions generally [123]. Other air pollutants can also adversely affect the brain during development. For instance, when silica and titanium dioxide NPs were injected intravenously to pregnant mice, they could be detected in the fetal brain [124]. This suggests that NPs can cross the maternal-fetal barrier in the placenta and could cause neurotoxicity in the offspring. Using an *ex vivo* human placental perfusion model, Wick et al. found that nanosized material can cross the placenta without affecting the viability of the placental explant *per se *[125]. However, the ability of ambient air pollutants to cross the placenta needs further evaluation to understand the full spectrum of possible effects.

Epidemiological and clinical studies demonstrating a negative impact of air pollution on neural development in humans were performed in children living in Mexico City [10, 11, 107]. 181 children of African-American and Dominican origin from New York City, who had valid prenatal polycyclic aromatic hydrocarbons (PAHs) monitoring data, were evaluated for mental and psychomotor development at age 3 [126]. Prenatal exposure to high concentration of PAHs was found to be associated with a lower mental development index at age 3. A second study from Boston, examined the relation between CB and cognition [127]. Long-term exposure to CB particles was associated with a decrease in cognitive testscores, even after adjustment for socioeconomic status, birth weight, smoking, and blood lead level. These studies, however, have certain limitations, such as limited monitoring of pollutants levels and significant reduction in the sample from the original cohort over time. Moreover, the presence of confounding factors was not addressed in these studies. A third study showed that early-life exposure to emissions from indoor gas appliances is negatively correlated with neuropsychological development through the first 4 years of life. Children that carried the glutathione-S-transferase gene Val-105 allele were particularly susceptible to the effects [128]. Electrophysiological examinations confirmed disturbances in brain development as a result of exposure to polluted air. Brainstem auditory-evoked potentials (BAEPs) were compared across children from highly and lowly polluted cities [129]. Children from the highly polluted environments displayed significant delays in the central conduction time of BAEPs, suggesting that exposure to air pollution may be a risk for auditory and vestibular impairment.

Prenatal exposure to air pollutants may also constitute a risk factor for neurodevelopmental disorders such as autism and neuropsychiatric diseases such as schizophrenia. Schizophrenia is a chronic disease of the brain that is characterized by positive and negative psychiatric symptoms as well as cognitive dysfunction. The incidence of schizophrenia in the population is about 1% [130]. Schizophrenia is caused through a combination of genetic factors and environmental insults, for instance prenatal infection [131]. An increased risk for schizophrenia is evident in people inhabiting urban regions [132]. The exact reasons remain unclear, but exposures to infectious agents or toxins from the urban environment have been suggested as possible causes. An important feature of airborne PM is that they may interact with other pathogens to serve as transporters for viruses, bacteria or molecules with infectious or antigenic properties, for instance, bacteria cell wall components [133]. Contrary results, however, were reported in a study from Finland, showing a correlation between living in rural regions and increased risk for schizophrenia [134]. The authors suggested that nontraffic source of air pollution, such as firewood, could have been a possible risk factor [134].

Autism on the other hand is a neurodevelopmental disorder that is characterized by impairments in social interaction, verbal and nonverbal communication, and repetitive behavior [135]. The prevalence for autism in the general population has been reported to range from 0.2 to 0.6% with an increasing trend over the recent years. Although, the exact etiology of autism is still unclear, genetic, environmental, and social factors may contribute to the development of the disease [136]. Maternal exposure to air pollution during the prenatal period may also be a risk factor. 284 autistic children and 657 healthy controls were examined in a San Francisco study that evaluated the possible effects of air pollution on autism development [137]. An association was found between the estimated concentrations of metals and solvents in the ambient air around the birth residence and autism. An association was also found between autism and residential proximity to freeways during the third trimester [138]. These alerting results suggest that subtle health effects, such as functional delays in brain maturation and impairment of neurobehavioral competences, should be included in studies of chronic effects of urban air pollution [116].

As derived from studies on the aged population, air pollution also has adverse effects on mental health during adulthood [113, 114]. A study by Chen and Schwartz demonstrated that neurobehavioral effects are associated with long-term exposure to ambient PM and ozone in adults [139]. Further longitudinal studies are urgently needed to fully explore the relationship between long-term exposure and neurobehavioral changes and subsequent development of neurocognitive impairment, such as cognitive decline and dementia. Ten human volunteers were exposed to dilute amounts of DEPs (300 *μ*g/m^3^), and brain activity was monitored by quantitative electroencephalography (EEG) showing a significant increase in the EEGs median power frequency and fast wave activity [140]. Additional studies need to determine whether other types of air pollutants elicit comparable effects on brain activity. The use of recent and more sophisticated technology, such as functional MRI and recording of event related potentials, in future studies will contribute to a better understanding of the relationship between air pollution and mental health.

## 5. Cellular and Molecular Mechanisms of Neuronal Injury Induced by Air Pollution

Air pollution can produce its adverse effects in the CNS through a variety of cellular and molecular mechanisms (Figure 1). Given the complex nature of polluted ambient air, CNS pathology is probably a result of the synergistic interaction of multiple pathways and mechanisms [1]. Although the exact mechanisms that are responsible for air pollution-induced neurotoxicity are poorly understood, postmortem and experimental studies suggest that air pollution causes oxidative stress, neuroinflammation, cerebrovascular damage, and cell death, which are also common features of neurodegenerative disorders. Genetic and epigenetic mechanisms might also be involved.

### 5.1. The Interaction of Air Pollutants with Cells and Cellular Organelles

Possible mechanisms by which air pollutants can interact with biological tissue depend on the size, the structure, and the composition of the components in the polluted air, determining their spectrum of molecular activity and entry routes. PMs can be taken up by mammalian cells in different ways, including phagocytosis, pinocytosis, passive diffusion, receptor-mediated endocytosis, direct penetration of the cell membrane, or transcytosis. Which route is taken largely depends on the physicochemical properties of the toxic components. PM that cannot enter cells directly could still interact with surface proteins and change cellular signaling and behavior.

There is a particular relationship between the particle size and the ways by which it can be taken up by cells. While the uptake of fine particles (0.1–2.5 *μ*m diameter) by macrophages is a specific receptor-mediated process (phagocytosis) the uptake of ultrafine particles (<0.1 *μ*m diameter) can occur by other, nonspecific mechanisms. These mechanisms may include electrostatic, van der Waals, and steric interactions and are subsumed under the term adhesive interaction, although the exact mechanisms remain to be determined [13, 33]. As mentioned before, ultrafine PMs can cross red blood cell membranes rapidly and easily; a process that appears to be mediated by an unidentified non-phagocytic mechanism [33]. Particles smaller than 100 nm could be observed in intraluminal erythrocytes that were collected from frontal lobe and trigeminal ganglia capillaries from postmortem brain tissue [10]. UFPs have a very large surface-to-volume ratio and are not enclosed by membranous organelles, which allow them to directly interact with intracellular proteins, organelles, or DNA. Such particles may reach specific organelles, such as mitochondria, lysosomes, and nuclei, where they could induce an oxidative burst within their membranes by interfering with NADPH-oxidase activity. They may also induce the release of inflammatory mediators and cytokines by the cell [13]. A recent study has shown that exposure to airborne UPMs is associated with mitochondrial damage, as reflected by an increase in the copy number of mitochondrial DNA (mtDNA) [141]. Damaged mitochondria may then contribute to increased oxidative-stress through altered ROS production and subsequently overloading the cell with ROSs, or by interfering with cellular antioxidant defense mechanisms.

Interaction of airborne PM with cellular proteins can also result in protein degradation and protein denaturation. Loss of enzyme activity and formation of autoantigens are possible consequences [142]. Environmental NPs can also significantly increase the rate of protein fibrillation, which provides a possible link between air pollution and neurodegenerative disorders [143, 144]. If these findings can be confirmed under realistic *in vivo* conditions, it would have far-reaching consequences with respect to the mechanisms underlying neurodegenerative diseases [12]. Other key molecular pathways that are affected in neurodegenerative diseases lead to misfolding, aggregation, and accumulation of proteins in the brain [145, 146]. PMs that have the capability to enter nerve cells could contribute to these processes, so could oxidative stress that is induced by the air pollutants.

Cellular responses to oxidative stress can lead to changes in mitochondria and other organelles, notably the endoplasmic reticulum (ER), and eventually triggers the cell to enter a cell death pathway [147, 148]. Mitochondria, as regulators of cellular energy metabolism and apoptosis, are critical organelles in switching between different cellular responses leading to death or survival of the cell. Perturbed ER calcium homeostasis may also contribute to neuronal dysfunction and degeneration in neurodegenerative disorders [149]. The ER is critical for early protein biosynthesis steps of secreted and membrane proteins, which occurs in the lumen of the ER, where the ER machinery assists in their folding.

Loss of ER homeostasis triggers stress responses, which are a hallmark of many inflammatory and neurodegenerative diseases [150]. Recent studies have shown that exposure to airborne PM causes ER stress in lung tissue [151, 152]. Neurodegenerative disorders are often characterized by the aggregation and accumulation of misfolded proteins [153]. Protein folding stress in the ER may lead to activation of the unfolded protein response (UPR). Organic DEP chemicals induce an UPR and proinflammatory effects in human bronchial epithelial cell line [154]. However, the possible relationship between ER stress and exposure to air pollution has not been studied in the context of CNS cells. The interesting crosstalk between innate immune pathways and ER-signaling that regulates the intensity and duration of innate immune responses should also be considered in neuroinflammation-induced by air pollution [150].

### 5.2. Neuronal and Glial Cell Death

Air pollution-induced loss of neurons is a consistent finding in postmortem and experimental studies, and neuronal cell death may be direct or indirect via microglia activation. It is noteworthy that several different types of NPs, including ambient UFPs, target mitochondria directly [42, 142]. This can lead to disruption of the mitochondrial electron transport chain, which leads to increased superoxide radical production. Furthermore, ambient UFPs perturb the permeability of the mitochondrial transition pore, resulting in the release of proapoptotic factors and ultimately programmed cell death [142]. It has also been suggested that presynaptic terminals are a target for NP-mediated changes in glutamatergic neurotransmission, which can result in neuronal damage and finally neurodegeneration [155].

In addition to neurons, other CNS cells may also be target of air pollution. Indeed, astroglial cell death has been reported upon exposure to high dose of ozone *in vitro *[48]. As suggested by MRI studies in dogs and children of Mexico City, oligodendroglial cells may be affected by air pollution [11, 80], and prefrontal white matter hyperintense lesions were observed in these studies. However, any experimental study specifically focusing on the effects of air pollution on oligodendrocytes and myelin has not been reported so far. Brain endothelial cells and pericytes are other candidate target cells. Exposure to DEPs resulted in endothelial activation and dysfunction in rat brain capillaries, but cell viability was not assessed in the study [47].

Besides apoptosis and necrosis, additional cell death mechanisms may also contribute to air pollution-induced CNS injury. Increased levels of autophagic vacuoles were observed upon exposure of cells to NMs *in vitro* [156]. Autophagy is a cellular process for the disposal of damaged organelles or denatured proteins through a lysosomal degradation pathway. The interaction of NMs with the autophagy pathway may be disruptive to neurons, leading to severe structural changes and ultimately cell death. Impaired autophagy is also implicated in the pathogenesis of neurodegenerative disorders [157]. However, the exact role of autophagy in CNS injury induced by air pollutants remains to be identified.

### 5.3. Oxidative Stress, DNA Damage, and Genotoxicity

Oxidative stress refers to an imbalance between the production of ROS and the cells ability to detoxify reactive intermediates or to repair cellular damage caused by ROS. They are highly reactive molecules because of their unpaired electrons and form as natural byproducts of a cell normal oxygen metabolism. They also fulfill important roles in cell signaling and homeostasis. However, during times of environmental stress such as air pollution, ROS levels can increase dramatically, resulting in significant damage to cellular components, including proteins, lipids, and DNA. Disturbances in the normal redox-state of tissues can cause toxic effects through the production of peroxides and free radicals (e.g., chemical species that contains one or more unpaired electrons). The two most important oxygen-derived free radicals are superoxide and hydroxyl radicals. Free radicals are important for a number of biological processes, such as the elimination of bacteria by phagocytic cells. Excessive ROS accumulation, however, poses a challenge for cell survival, and cells have developed defense mechanisms against excessive amounts of ROS that include antioxidant enzymes (superoxide dismutase, catalase, and glutathione reductase, glutathione peroxides) and antioxidant molecules (glutathione, taurine, selenium, vitamins E and C).

Under normal conditions, ROS are generated at low concentrations and are easily neutralized by cellular antioxidant defenses such as glutathione (GSH) and antioxidant enzymes [142]. However, under conditions of excess ROS production, antioxidant and detoxification enzymes (phase II enzymes) are induced. The expression of genes that encode these enzymes contain antioxidant response elements (ARE) in their promoter regions, which contains a binding site for the nuclear factor (erythroid-derived 2)-like 2 (Nrf2) transcription factor [158]. At moderate levels of oxidative stress, the Nrf2 protective response pathway is activated; resulting in mitogen-activated protein kinase- (MAPK) and NF*κ*B- (a redox-sensitive transcription factor) induced proinflammatory responses [142]. Increased intracellular calcium levels also mediate the activation of these signaling pathways. At high levels of oxidative stress, perturbation of the mitochondrial permeability transition pore and the electron transfer chain cause apoptotic and necrotic cell death. Nrf2 regulates the expression of numerous cytoprotective genes that function to detoxify reactive species produced during ambient air pollutant metabolic reactions, highlighting the important role of Nrf2 in the defense against air pollutant-induced toxicity [158]. Dysfunction of Nrf2 may also be a risk factor for neurodegenerative diseases such as PD [159]. However, the possible role of Nrf2 in air pollution-induced injury has not yet been studied in the context of CNS.

The brain is especially vulnerable to oxidative stress injury because of its high metabolic activity, its low activity of antioxidant enzymes (superoxide dismutase and catalase), its low content of endogenous radical scavengers, such as vitamin C, its high cellular content of lipids and proteins, and its high amounts of redox metals such as iron and copper which can act as a potent catalyst for ROS production [103, 160]. Oxidative stress has been consistently linked to aging-related neurodegenerative diseases leading to the generation of lipid peroxides, carbonyl proteins, and oxidative DNA damage in tissue samples from affected brains [103, 161]. Metals, pesticides, and air pollutants, all of which have been associated with neurodegeneration share a common feature, namely, their capacity to lead to increased production of reactive oxygen and nitrogen species. Although each pollutant has its own mechanism of toxicity, several air pollutants, like ozone, sulfur dioxide, volatile organic compounds, and PM, are oxidants that can act directly on cellular components and disturb physiological functions [17, 162–164]. Some of these pollutants go through a series of metabolic reactions catalyzed by phase II enzymes, in order to be detoxified and excreted. These reactions involve chemical modifications, like oxidation, to increase the solubility of the original compound so that it can be excreted. During these metabolic reactions, many reactive intermediates, particularly ROS, are produced [158]. Both postmortem and *in vivo* studies have recently revealed a link between oxidative stress and air pollution-induced CNS injury [10, 47, 71, 73, 78]. For toxicological screening studies, more refined approaches, for example, the use of nanosensors to detect ROS generation by NPs will emerge with time [32, 142].

Exposure to combustion particles is consistently associated with oxidative damage to DNA and lipids in humans detected from leukocytes, plasma, urine, and exhaled breath [165, 166]. The evaluation of apurinic/apyrimidinic sites in nasal and brain genomic DNA in healthy dogs naturally exposed to urban pollution in Mexico City showed DNA damage suggesting a link to air pollution [39]. DNA damage is also crucial in aging and in age-related disorders, such as AD. The processes involved in particle-induced genotoxicity remain poorly understood, because the particles are uniquely complex and of diverse physicochemical characteristics [16]. Interestingly, a recent study evaluating the link between gaseous air pollutants and brain cancer mortality did not provide evidence for an increased risk of mortality due to air pollution [167].

### 5.4. Microglial Activation

Microglia, the macrophage-like cells of CNS, are the principal players in the brain's innate immune response. They are the immunocompetent cells of the brain that continuously survey their environment with highly motile extensions [168]. Microglial cells normally provide tissue maintenance and immune surveillance to the brain and exert a neuroprotective role by their ability to phagocytose aggregated disease proteins and pathogens and to secrete neurotrophic factors. Microglia cells rapidly change their cell morphology in response to any disturbance of nervous system homeostasis and are then referred to as activated on the basis of morphological changes and expression of cell surface antigens [168]. Microglial activation is the main cellular event during neuroinflammation. The activation of microglia results in the production and release of a myriad of inflammatory cascade mediators, including Nitric oxide (NO), chemokines, proinflammatory cytokines, ROS, and reactive nitrogen species (RNS) those are deleterious to the CNS [169]. Microglial activation and inflammation are also associated with progressive neuronal apoptosis in human neurodegenerative diseases [170–172]. However, it is not clear whether activation of microglia and the inflammatory responses play a role in the cause of the disease or whether cell activation is a response to the early changes associated with the disease process.

Microglia are also activated in response to aggregated disease proteins (A*β* and *α*-synuclein), bacterial endotoxins (LPS), proinflammatory cytokines, MMP-3 released from apoptotic neurons, and environmental neurotoxins [1, 173]. An important molecular component of microglial responses is the toll-like receptor 4 (TLR4), a pathogen-receptor known to initiates an inflammatory cascade in response to various CNS stimuli [174]. LPS, as the prototypical endotoxin, binds to a CD14/TLR4/MD2 receptor complex and enables TLR4 signaling. Human autopsy studies showed evidence for increased CD14 expression in response to chronic exposure to high levels of air pollution, indicating an activation of either infiltrating monocytes or the resident microglial cells [112]. Similar findings were observed in brain tissue of young healthy dogs exposed to air pollution [80]. As demonstrated by morphological changes and increased superoxide production in a neuron-glia cell culture system, DEPs can also activate microglia *in vitro *[46]. Furthermore, neuron-glia cocultures treated with DEP showed selective dopaminergic neurotoxicity that only occurred in the presence of microglia, indicating that activated microglia cells mediate the neuronal damage. Neuron-glia co-cultures derived from mice lacking functional NADPH oxidase, the enzyme responsible for extracellular superoxide production, were insensitive to DEP-induced neurotoxicity, indicating that microglia-derived ROS mediate DEP-induced dopaminergic neurotoxicity [1, 46]. Interestingly, cytochalasin D, a phagocytosis inhibitor, reduced DEP-induced superoxide production in enriched-microglia cultures, implying that DEP is phagocytized by microglia to trigger the production of superoxide [46], whereas UFPs themselves can inhibit phagocytosis in alveolar macrophages [175]. This difference may result from the differences in cell or particle type. A very recent *in vivo* study could also demonstrate DEP-induced microglial activation, neuroinflammation, and dopaminergic neurotoxicity [49].

Metals associated with air pollution are also able to activate microglia. Manganese, a component of industrial-derived air pollution, is able to activate rat microglia *in vitro *[176]. Microglial activation by manganese chloride also induces dopaminergic neurotoxicity *in vitro* and application of antioxidants, such as superoxide dismutase/catalase, glutathione, NAC, or inhibitors of NO biosynthesis significantly protected dopaminergic neurons against damage [177]. LPS on the other hand amplifies neurotoxicity induced by activated microglia in response to manganese chloride [178]. Interestingly, the responses of microglia and astroglia to these activators differ, although both cell types are regarded as cellular components of the brain's innate immune system.

### 5.5. Neuroinflammation and Inflammasome Activation

Neuroinflammation is a complex and innate response of neural tissue against harmful stimuli such as pathogens, damaged cells, and other irritants within the CNS. A crucial component of innate immunity in the CNs involves the production of proinflammatory cytokines mediated by inflammasome signaling [179]. The innate immune cells in the CNS, microglia and astrocytes, express pattern-recognition receptors (PRRs), for example, TLR4, which participate in the assembly and activation of the inflammasome [179]. The inflammasome itself is a multiprotein complex that consists of caspase 1, PYCARD, NALP (a NOD-like receptor serving as a PRR), and sometimes caspase 5 or caspase 11 [180]. Nucleotide-binding domain, leucine-rich repeat, pyrin domain containing 3 (NLRP3) are a key component of the inflammasome complex, which also includes ASC (apoptotic speck-containing protein with a card) and procaspase-1 [181]. The exact composition of the inflammasome depends on the activator which initiates its assembly, that is, dsRNA will trigger one inflammasome composition, whereas asbestos will induce the assembly of a different variant. The inflammasome promotes the maturation of inflammatory cytokines such as IL-1*β* and interleukin 18 (IL-18). It has also been shown to induce cell pyroptosis, a process of programmed cell death that is distinct from apoptosis [181]. The inflammasome orchestrates the activation of caspase precursors, which in turn, cleave the precursor forms of the cytokines as IL-1*β*, IL-18 and interleukin-33 (IL-33), which triggers an inflammatory response, or the release of toxins from glial and endothelial cells [179].

Inflammasome activation was recently shown to be induced in acute brain injury as well, thus the NLRP1 inflammasome may constitute an important component of the CNSs' response to traumatic brain injury [182]. An inflammasome complex also forms after experimental focal brain ischemia as could be demonstrated by immunohistochemical analysis of inflammasome proteins in neurons, astrocytes, microglia, and macrophages [183]. The NLRP3 inflammasome also plays an important role in an experimental model of MS, which is mediated by caspase-1 and IL-18 [184]. Although it has recently been shown that the NALP3 inflammasome is involved in the innate immune response to A*β* in microglia [185], the specific pathophysiologic role of the inflammasome in neurodegenerative disorders still remains to be clarified [186].

The organic substances adsorbed onto airborne Asian sand dust activate the NALP3 inflammasome in macrophage cell lines and murine lung [187]. Exposure of macrophages to CB induces inflammasome activation and pyroptosis [188]. The identification of pyroptosis as a cellular response to carbon NP exposure is novel and has important consequences for environmental and health-related issues. Another study showed that TiO_2_ and SiO_2_ NPs activate the NLRP3 inflammasome in cultured keratinocytes, murine lung, and dendritic cells [189, 190]. Whether air pollutants induce inflammasome activation in CNS and neuroglial cells remains to be identified.

### 5.6. Reactive Astrogliosis

Astrocytes are characteristic star-shaped glial cells that outnumber neurons in the brain about fivefold. They perform many functions, including biochemical support of cerebral endothelial cells that form the BBB, provision of nutrients to the nervous tissue, maintenance of extracellular ion balance, buffering of excess neurotransmitters, secretion of neurotrophic factors, control of cerebral blood flow, supporting neurogenesis as well as repair of injured brain and spinal cord [191]. Reactive astrogliosis is a ubiquitous feature of CNS pathologies [192]. At later stages of CNS disorders, astrocytes become activated and contribute to neuroinflammation and neurodegeneration. Astroglia were reported to be activated in humans that were chronically exposed to high levels of air pollution, as evidenced by enhanced glial fibrillary acidic protein (GFAP) expression [9, 10]. Animal studies investigating ozone exposure showed that astroglial cells that are located close to brain capillaries have enhanced expression of IL-6 and TNF*α* [79] or are increased in number [193]. However, it is unclear whether the astroglia respond to components of air pollution, to the inflammation, and oxidative stress produced from other cell types or to cellular damage [1].

### 5.7. Impacts on the Blood-Brain Barrier

The BBB is the major site of controlled blood-CNS exchange. This physical barrier protects the CNS from potential toxins and pathogenic agents. An intact BBB is important for the proper functioning of the CNS by actively controlling cellular and molecular trafficking between the systemic circulation and the brain parenchyma [194]. Cerebral endothelial cells have luminal tight junctions that form the physical barrier of the inter-endothelial cleft. Endothelial cell are covered on the outside by a basement membrane, which also surrounds pericytes. Around these structures end-feet processes from nearby astrocytes can be found which seal the BBB additionally [195]. The BBB integrity is impaired in many common CNS disorders such as AD, PD, and stroke [196]. Activation or damage of the various cellular components of the BBB facilitates leukocyte infiltration leading to CNS injury. Systemic inflammation induced by inhaled air pollutants can disturb the integrity of the BBB through the effects of circulating proinflammatory cytokines and LPS on cerebral endothelial cells [1]. Furthermore, an increase in ROS is a common trigger for many downstream pathways that directly mediate BBB compromise such as oxidative damage, tight junction modification and matrix metalloproteinases (MMP) activation [197]. Air-borne particulate matter has been identified both in human brain capillaries and in the brain parenchyma, although the exact transport mechanisms are unclear [10]. Additionally, increased expression of intercellular adhesion molecule (ICAM) and vascular cell adhesion molecule (VCAM) was observed in cerebral vasculature suggesting endothelial activation. As demonstrated by an *ex vivo *study, DEPs induce oxidative stress, proinflammatory signaling, and P-glycoprotein up-regulation in the rat brain capillaries [47]. These findings suggest that the BBB is an important target for air pollutants. Therapeutic strategies that aim to change BBB permeability may combat neurotoxic effects of air pollutant on the CNS.

### 5.8. Gene-Air Pollution Interaction and Epigenetic Mechanisms

Individual differences that were observed upon exposure to the same polluted ambient air suggest that genetic susceptibility is likely to play a role in response to air pollution [198]. Gene-air pollution interaction was extensively studied in pulmonary and cardiac disorders [198, 199]. There are, however, only a limited number of studies that address gene-air pollution interaction in CNS injury. The Apo *ε*4 allele shows an amplifier effect on brain injury caused by exposure to air pollution [107]. Air pollution-induced olfactory dysfunction, also an early indicator for neurodegeneration, was higher in Apo *ε*4 carriers [110] and experimental *in vivo *studies showed that APOE^−/−^ mice were more vulnerable to neuropathology induced by air pollution [66–68].

Another susceptibility gene for the effects of air pollution in the brain may be the glutathione-S-transferase gene (GSTP1) because of its important role as radical scavenger [128]. Adverse effects of exposure to nitrogen dioxide on cognitive function are more significant in children with any GSTP1 Val-105 allele. Since oxidative stress, and inflammatory processes are common denominators of air pollution-induced neuropathology, oxidative stress and inflammatory pathway genes including Glutathione S-transferase Mu 1(GSTM1), GSTP1, NAD(P)H dehydrogenase quinone 1(NQO1), TNF, and TLR4 are further logical candidates for the study of the association with the susceptibility to air pollutants [200].

Air pollutants can change gene expression through a broad array of gene regulatory mechanisms. Epigenetics is a posttranscriptional control mechanism in gene regulation. Changes in DNA methylation and histone acetylation leads to imprinting, gene silencing, and suppression of gene expression without altering the sequence of the silenced genes [201]. Epigenetic alternations are often involved in the pathogenesis of neurological disorders [202, 203]. Air pollution related neurological damage may occur via epigenetic effects and could be demonstrated [204, 205]. Nano and microsized SiO_2_ exposure significantly decreased genomic DNA methylation and levels of the related methyl transferase in normal HaCaT epithelial cells line [205]. DEP exposure induces Cox-2 gene expression by increasing histone H4 acetylation and histone deacetylase 1 (HDAC1) degradation in bronchial epithelial cells [204]. Similar results were also obtained from *in vivo* studies [206]. Exposure of inbred mice to particulate air pollution caused hypermethylation in spermatogonial stem cells. Human studies showed that either short- or long-term exposure to air pollution in elderly can cause hypomethylation in peripheral lymphocytes [207, 208]. In addition, higher exposure to traffic-related air pollution is associated with shorter leukocyte telomeres, which is a sign of biological aging [209]. Further studies are necessary to clarify in how much epigenetic changes contribute to neurological symptoms caused by air pollution.

## 6. Conclusions and Future Prospects

Air pollutants have been, and continue to be, major contributing factors to chronic diseases and mortality, thereby dramatically impacting public health. Air pollution is a global problem and has become one of the major issues of public health as well as climate and environmental protection. The effects of air pollutants are thus at a high level of interest for scientific, governmental, and public communities. An increasing number of people are exposed to a complex mixture of inhalable NPs and toxic chemicals occupationally or as a result of man made and natural disasters, such as war, fires, and volcanic eruptions [210, 211]. Air pollution is increasingly recognized as an important and modifiable determinant of cardiovascular and respiratory diseases in urban communities [3, 16]. Although adverse cardiopulmonary outcomes have been the focus of many studies, air pollution-related damage to the CNS has been widely neglected. However, there is mounting evidence that air pollution also contributes to CNS damage or increased progression of neurodegenerative disorders.

The data discussed as part of this critical update highlight that UFPs rapidly translocate from the lungs into the cells and into the blood circulation. There is good evidence that oxidative stress occurs in other organs, such as the heart and the brain. The breadth, strength, and consistency of the preclinical and clinical evidence provide a compelling argument that air pollution, especially traffic-derived pollution, causes CNS damage and that there is a clear link between air pollution and neurological diseases. Airborne particles cause neuropathology, which seem to be mediated by direct or indirect proinflammatory and oxidative responses. Both, the physical characteristics of the particle itself and toxic compounds adsorbed on the particle may be responsible for the damage. The time of exposure has a key role in damage. Minimum doses of pollution can be handled by the organism when this exposure is acute, but the same doses administered chronically lead to an oxidative stress state that can produce neurodegeneration. Astroglia, cerebral endothelial cells, and microglia in particular respond to components of air pollution with chronic activation, inflammation, and oxidative stress [1]. CNS effects can be chronic, can begin in early childhood, and may accumulate with age [1].

Given the enormous complexity of the CNS and the complex nature of air pollution, the resulting CNS pathology can have many underlying causes and pathways and could be due to synergistic interaction of multiple pathways and mechanisms making it difficult to pinpoint a clear stimulus-response relationship. While epidemiological data link increased risk for stroke, MS, and PD to the exposure to specific air pollutants, further experimental and mechanistic studies aiming at the association between the components of air pollution and the development of CNS diseases are of pressing importance for mental health [1]. The adverse effects of the complex mixtures of polluted air components are poorly understood. For instance, the contribution of direct effects of airborne UFPs to CNS injury remains to be worked out in detail, and data on the presence of UFPs in the human CNS are still lacking to date. The biological studies can be strengthened by the use of recent discovery tools and platforms, such as proteomics and genomics, to develop biomarkers for toxicity screening [142]. The main problems that are encountered in testing air pollutants toxicity in humans are dosimetry, the lack of appropriate standardized protocols, and good quantitative descriptions of real-world exposure conditions [60, 142]. Novel detection methods need to be developed for exposure assessment and dosimetry calculation.

Our current knowledge provides a basis for much more extensive epidemiological, forensic, and toxicological studies aimed at identifying the underlying mechanisms of neural damage, and strengthening of the association between chronic exposure to air pollutants, and the risk of developing neurological diseases. However, epidemiologic and observational data are limited by imprecise measurements of pollution exposure, the potential of environmental, and social factors to confound the apparent associations. Since genetic susceptibility is likely to play a role in response to air pollution, gene-environment interaction studies can be a tool to explore the mechanisms and the importance of molecular pathways for the association between air pollution and CNS damage [198]. Inconsistencies between studies sometimes prevent us from drawing firm conclusions. The limited sample size of most studies, difficulty in quantifying exposure, providing a qualitative description of active components from complex environmental air samples, method of ascertainment, time of measurement, and collinearity between pollutants make difficult to use for the study of gene by gene interactions [200]. More studies and more intensive collaborationsare needed to generate larger and more diverse cohorts and standardized data that would allow us to draw stronger conclusions [198]. The roles of gene-air pollution interactions and epigenetic mechanisms need to be considered [200]. Better understanding of the mediators and mechanisms of CNS injury due to air pollution will help to develop preventive and treatment strategies for the protection of individuals at risk. Improving air quality standards, minimizing personal exposures, and the redesign of engine and fuel technologies will also reduce air pollution and its consequences for neurological morbidity and mortality.

## Figures and Tables

**Table 1:** The effects of air pollutants on neuronal and glial cells *in vitro*.

Pollutants	Species	Cell type	Assays	Key findings	References
PMs					
DEPs	Rat	VM Neuron-glia	TH immunostaining	DA neurotoxicity	[46]
			OX-42 immunostaining	Microglial activation	
	Mouse (PHOX^−/−^)	Microglia	DCFH-DA	Increased microglial ROS	
		Microglia	DA uptake	No DEP neurotoxicity	
DEPs + LPS	Rat	VM Neuron-glia	Griess reaction	Increased NO production	[49]
	Rat	HAPI microglial	TH immunostaining	DA neurotoxicity	
		cell line	ELISA	Increased TNF*α* release	
DEPs	Rat	Brain capillaries	CM-H_2_DCFDA	Increased ROS production	[47]
			ELISA	Increased TNF*α* release	
CAPs	Mouse	BV2 microglial cell line	ELISA	Increased TNF*α*, IL6 release Increased P-glycoprotein	[50]
			Western blotting microarray	Upregulated inflammatory genes	

Ozone	Rat	Astrocyte	MDA	Increased lipid peroxidation	[48]
			LDH	Decreased cell viability	

Abbreviations: concentrated ambient particles (CAPs), 2′,7′-dichlorfluorescein-diacetate (DCFH-DA), dopaminergic (DA), diesel exhaust particles (DEPs), enzyme-linked immunosorbent assay (ELISA), interleukin-6 (IL-6), lactate dehydrogenase (LDH), malondialdehyde (MDA) nitric oxide (NO), reactive oxygen species (ROS), tyrosine hydroxylase (TH), tumor necrosis factor alpha (TNF*α*), ventral mesencephalic (VM).

**Table 2:** The effects of air pollutants on the central nervous system *in vivo. *

Pollutants	Species	Route of administration	Assays	Key findings and outcome	References
PMs					
	Mouse (ApoE^−/−^)	Inhalation	TH immunostaining	DA neurotoxicity Astrogliosis	[66]
CAPs	Mouse (ApoE^−/−^)	Inhalation	EMSA Western blotting	NF*κ*B and AP-1 activation JNK activation	[67]
	Mouse	Inhalation	EMSA ELISA	NF*κ*B activation	[68]

				Increased TNF*α* and IL-1 *α* levels
DEPs	Rat	Inhalation	IHC, RT-PCR Western blotting	Increased HO-1 and COX2 mRNA and protein expression	[69]
	Mouse	Inhalation (w/wo i.p. LTA injection)	Microdialysis/HPLC RT-PCR	Increased glutamate levels Inreased NMDA receptor subunits (NR1, NR2A, and NR2B), and CaMKIV mRNA	[70]

	Inhalation (nasal)		EMSA ELISA, RT-PCR	NF*κ*B and AP-1 activation Increased TNF*α* and IL-1 *α* levels and mRNA expression	[71]
Rat	Mice	Inhalation	Open-field test	Decreased locomotor activity	[72]
	Rat	Inhalation/Intratracheal	IBA-1 immunostaining	Microglial activation	
			ELISA	Increased TNF*α*, IL-1*β*, IL-6, and MIP-1*α* levels	[49]
			qRT-PCR	Increased TNF*α*, MIP-1*α* mRNA expression	

ROFA	Rat	In instillation	TBARs	Increased lipid peroxidation	[73]

			Open-field test	Decreased exploratory behavior
NSCB	Mouse	in instillation (w/wo ip LTA injection)	RT-PCR Microdialysis/HPLC	Increased TNF*α*, IL-1*β*, and chemokine mRNA expression Increased glutamate glycine levels	[74]

	Rat	Inhalation (temporal)	Motor activity test Lipid peroxidation assay TH immunostaining	Decreased motor activity Increased lipid peroxidation Loss of DA neurons (SN)	[75]
	Rat (ovariectomized)	Inhalation	TH immunostaining	Loss of DA neurons (SN)	[76]
	Rat	Inhalation	Behavioral tests	Increased freezing behavior Decreased exploratory behavior	[77]
			Lipid peroxidation assay	Increased lipid peroxidation	
				Neurodegeneration	
			Electron microscopy Microdialysis/HPLC	Changes in neurotransmitter levels	
Ozone	Rat(ovariectomized)	Inhalation	Behavioral tests Lipid peroxidation assay	Impaired olfactory perception and social recognition memory Increased lipid peroxidation	[71]
	Rat	Inhalation	Behavioral tests Lipid peroxidation assay	Impaired memory Increased lipid peroxidation	[78]
	Rat	Inhalation	IHC	Increased VEGF, IL-6 and TNF*α*	[79]
	Rat	Inhalation	IHC	Increased c-Fos expression in different brain regions including NTS	[43]

Abbreviations: apolipoprotein E (ApoE), calcium/calmodulin-dependent protein kinase type IV(CaMKIV), concentrated ambient particles (CAPs), cyclooxygenase-2 (COX-2), dopaminergic (DA), diesel exhaust particles (DEPs), electrophoretic mobility shift assay (EMSA), enzyme-linked immunosorbent assay (ELISA), heme oxygenase (HO), high-performance liquid chromatography (HPLC), allograft inflammatory factor 1(IBA1), immunohistochemistry (IHC), interleukin-1 alpha (IL-1*α*), interleukin-1beta (IL-1*β*), interleukin-6 (IL-6), c-Jun N-terminal kinases (JNK), macrophage inflammatory proteins (MIPs), nanosized carbon black (NSCB), lipoteichoic acid (LTA), nuclear factor kappa B (NF*κ*B), nucleus tractus solitarius (NTS), residual oil fly ash (ROFA), reverse transcription polymerase chain reaction (RT-PCR), substantia nigra (SN), thiobarbituric acid-reactive substances (TBARS), tyrosine hydroxylase (TH), tumor necrosis factor alpha (TNF*α*), and vascular endothelial growth factor (VEGF).

## Abbreviations

A*β*: Amyloid-beta
AD: Alzheimer's disease
ARE: Antioxidant response element
ApoE: Apolipoprotein E
ASC: Apoptosis-associated speck-like protein containing a CARD
BAEPs: Brainstem auditory evoked potentials
BBB: Blood-brain barrier
CB: Carbon black
CNS: Central nervous system
CAPs: Concentrated ambient air particles
COX-2: Cyclooxygenase-2
DEPs: Diesel exhaust particles
EEG: Electroencephalography
ER: Endoplasmic reticulum
ENPs: Engineered nanoparticles
GFAP: Enhanced glial fibrillary acidic protein
GSH: Glutathione
GSTP1: Glutathione-S-transferase gene
GSTM1: Glutathione S-transferase Mu 1
HDAC1: Histone deacetylase 1
iNOS: Inducible nitric oxide synthase
IL-6: Interleukin-6
IL-1*β*: Interleukin-1beta
IL-18: Interleukin 18
ICAM: Intercellular adhesion molecule
LTA: Lipoteichoic acid
MMP: Matrix metalloproteinases
MCAO: Middle cerebral artery occlusion
mtDNA: Mitochondrial DNA
MAP: Mitogen-activated protein
MAPK: Mitogen-activated protein kinase-
MS: Multiple sclerosis
LPS: Lipopolysaccharide
NQO1: NAD(P)H dehydrogenase quinone 1
NMs: Nanomaterials
NP: Nano-sized particulate
NAC: N-acetylcysteine
NFTs: Neurofibrillary tangles
NO: Nitric oxide
NF*κ*B: Nuclear factor kappa B
Nrf2: Nuclear factor (erythroid-derived 2)-like 2
NLRP3: Nucleotide-binding domain, leucine-rich repeat, pyrin domain containing 3
NTS: Nucleus tractus solitarius
PRRs: Pattern Recognition Receptors
PD: Parkinson's disease
PM: Particulate matter
PAHs: Polycyclic aromatic hydrocarbons
RNS: Reactive nitrogen species
ROS: Reactive oxygen species
ROFA: Residual oil fly ash
WHO: World Health Organization
TLR4: Toll-like receptor 4
TNF*α*: Tumor necrosis factor alpha
UFPs: Ultrafine
UPR: Unfolded protein response
VEGF: Vascular endothelial growth factor
VCAM: Vascular cell adhesion molecule
VLM: Ventrolateral medulla.
